# Let It Chill: The Role of Interventional Radiology in Complicated Appendicitis. A Case Report

**DOI:** 10.7759/cureus.4908

**Published:** 2019-06-16

**Authors:** Aldo Javier Vázquez Mézquita, Estefanía Murrieta Peralta, Jorge Ramírez Landero, Giselle Andrea Uribe Campo, Manuel Guerrero Hernández

**Affiliations:** 1 Radiology and Molecular Imaging, The American British Cowdray Medical Center, Mexico City, MEX; 2 Anesthesiology, The American British Cowdray Medical Center, Mexico City, MEX

**Keywords:** complicated appendicitis, interval appendectomy, percutaneous drain, interventional radiology, abscess

## Abstract

Appendicitis is a common cause of acute abdominal syndrome that affects different group ages. In some cases, complications such as abscess formation or perforation may make taking an immediate surgical approach difficult. We report a case of a 39-year-old male who presented with appendicitis, with the presence of a well-circumscribed abscess. The surgeons and interventional radiologists at our institution preferred a conservative approach by placing ultrasound-guided percutaneous drainage and performing an interval appendectomy weeks after the initial symptoms. Through the presentation of this case, we want to make physicians, mostly in developing countries, aware of the benefits of interventional radiology in the management of complicated appendicitis.

## Introduction

Appendicitis is one of the most common surgical emergencies, with an incidence of one person per 1000 each year, and according to guidelines of intra-abdominal infections, it is the most common cause of intra-abdominal sepsis [1-2]. These are the reasons why radiologists should be aware to make a prompt diagnosis and include potential complications in the report.

Complications may include abscess formation, perforation, and bowel obstruction among others [3]. One of the most common complications is perforation, which should be immediately sealed or it may become a phlegmon [4]. These are traditionally treated with surgery, but nowadays, there has been a special interest in resolving them in another way in order to have less technical problems during surgery and less morbidity [3].

Originally, interval appendectomy was offered to those patients who underwent conservative treatment and had an appendiceal abscess associated [2]. However, together with antibiotic therapy and the placement of a percutaneous drain, interval appendectomy is an alternative to surgery during the initial phase [3-6].

In this case report, we present a conservative approach by interventional radiology in a patient with complicated appendicitis. The purpose of this case is to make physicians, mostly in developing countries, aware of the benefits of interval appendectomy.

## Case presentation

**Figure 1:**
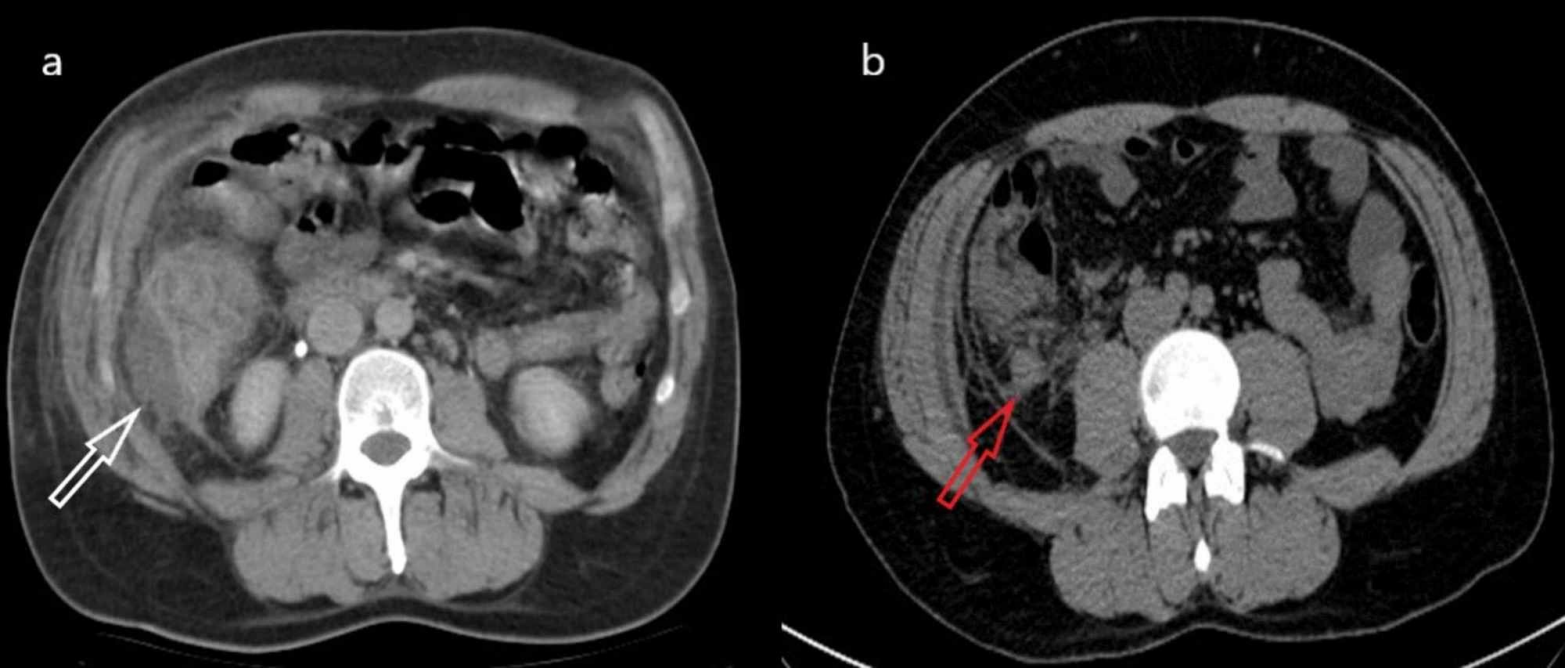
a) Axial non-contrasted abdominal CT scan, showing a collection adjacent to the ascending colon signaled by the white arrow; notice the surrounding stranded fat. b) Axial image of the same CT scan, with the red arrow showing an enlarged appendix with surrounding inflamed fat.

A 39-year-old male patient was received at our emergency department due to unspecified abdominal complaints and fever in the last four days. The patient denied any kind of pre-existing conditions. During the physical examination, the abdomen was soft, without any focal painful site. Blood laboratory tests showed marked leukocytosis (17 x 109/L), as well as a high neutrophil (15.6 x 109/L) count. A computed tomography (CT) scan was requested by the emergency physician, which showed a rounded hypodense structure located at the end of the appendix, adjacent to the ascendent portion of the colon. This structure showed higher attenuation with intravenous contrast material and stranded circumferential fat (Figure 1), which corresponded to an abscess. The appendix showed a diameter of 2 cm.

**Figure 2:**
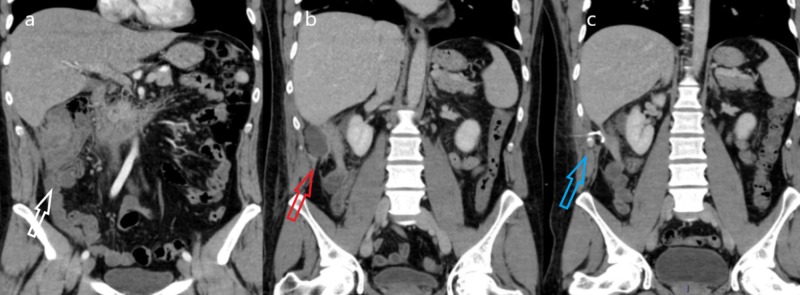
Coronal IV-contrast abdominal CT scans. a) Image shows the cecum with circumferential stranded fat signaled by the white arrow, notice the ascended position of the cecum. b) The red arrow signals a hypodense liquid-attenuated collection adjacent to the cecum. c) This image corresponds to the CT scan after drain placement signaled by the blue arrow, notice the volume reduction of the collection. IV: intravenous

Antibiotic and analgesic treatment was initiated because of the potential risk of complications. The case was presented to the interventional radiology department, which decided that a conservative approach was the best option for the patient. An ultrasound-guided percutaneous drain was placed in the right flank without any complications (Figure 2). After 11 days, the leukocyte count was normal (7000 x 109/L) and the patient was afebrile, therefore, the drain was taken out and the patient was discharged without any complications. An appendectomy was performed 14 days later, without any complications. The pathology report concluded inflammatory infiltrates secondary to appendicitis.

## Discussion

A non-surgical initial approach for complicated appendicitis has been questioned in the last decades. The purpose of it is to reduce the complication rate related to surgery, such as recurrent abscess and peritonitis, which could result in hemicolectomy. This initial non-surgical method is a well-established option for patients with comorbidities; nevertheless, in recent studies, it has been explored in different adults and children [4,6-7].

In the case of abscess formation, it has been discussed if imaging-guided drain placement is the best alternative, with fewer complication rates, and some articles consider it the first line of therapy [5]. In the Marin study [1], they reported a drain placement success rate of 90%, with a complication rate of 6% in adults [8]. The percutaneous drainage lasts around six days and the criteria for its removal are drain output less than 20 ml per day, fever resolution, and leukocytosis normalization [5,9]. However, it is noted that a control CT scan after drain placement is necessary in order to discard persistent or newly developed abscesses [1,2,5].

Interval appendectomy takes place around 62 days after the initial presentation, and it is considered a good alternative if malignancy is suspected, which has an incidence of 6% in adult patients with complicated appendicitis [5,9]. Nonsurgical treatment based only on antibiotics has been explored in the past; however, this conservative management has a high percentage of recurrent appendicitis between 10% and 25%, therefore, an interval appendectomy after antibiotic therapy is recommended [9].

There are some cases of complicated appendicitis in which early surgery is considered a better alternative, for example, the presence of an appendicolith because of bacterial colonization [10]. Another predictor of unfavorable clinical outcomes of percutaneous placement is the presence of a poorly defined abscess [6,8].

Regarding our case, the patient did not present any signs of instability that required an immediate appendectomy and the pericecal abscess was well-circumscribed. Therefore, percutaneous drain placement, as well as an interval appendectomy, were a good alternative in this particular case.

We acknowledge that this is a common approach in developed countries, however, immediate appendectomy is still regarded as the best option in developing countries for complicated and uncomplicated appendicitis [9-11]. Nonetheless, a systematic review by Cheng in 2017 concluded that there is not enough evidence to determine which of both approaches, immediate or interval appendectomy, has a lower complication rate [7]. We want to make aware more physicians that percutaneous drainage placement by interventional radiology and an interval appendectomy is a good option with a low complication rate for patients with complicated appendicitis.

## Conclusions

Interval appendectomy is a suitable alternative for cases of complicated appendicitis. A multidisciplinary approach is imperative in order to select the best therapy to reduce the complication rate and time of hospitalization.
